# Unusual clinical presentation of primary aortoduodenal fistula

**DOI:** 10.1093/gastro/gou040

**Published:** 2014-06-30

**Authors:** Daniele Bissacco, Luca Freni, Luca Attisani, Iacopo Barbetta, Raffaello Dallatana, Piergiorgio Settembrini

**Affiliations:** Vascular Surgery, Ospedale San Carlo Borromeo, Milan, Italy

**Keywords:** aorto-enteric fistula, aortoduodenal fistula, gastro-intestinal bleeding, abdominal aortic aneurysm, abdominal pulsatile mass

## Abstract

Primary aorto-enteric fistula (PAEF) develops between the native aorta and the gastro-intestinal tract, in the presence of an abdominal aortic aneurysm. It is a rare, life-threatening condition and appears to be less frequent than secondary aorto-enteric fistula, which is associated with previous aortic prosthetic reconstruction. When untreated, the overall mortality rate is almost 100%. Diagnosis may be challenging until the occurrence of a massive haemorrhage. In the presence of gross contamination, patients tend to a worse prognosis. Extra-anatomical bypass and repair of the enteric tract is the treatment of choice in case of gross contamination. *In situ* reconstruction is often reported in cases of mild bacterial contamination. Endovascular treatment has recently become a valid option in haemodynamically unstable patients, but a staged approach, with delayed surgical treatment, seems advisable.

## INTRODUCTION

Primary aorto-enteric fistula (PAEF) is a rare, life-threatening condition that develops between the native aorta and the gastro-intestinal (GI) tract, mainly in presence of an abdominal aortic aneurysm (AAA). PAEF is less frequent than secondary aorto-enteric fistula (AEF), which occurs in patients who have had previous aortic prosthetic reconstruction. A timely and accurate diagnosis may be challenging; insidious episodes of GI bleeding are frequently under-diagnosed until the occurrence of massive haemorrhage. When left untreated, its overall mortality rate is almost 100% [1]. In the presence of gross contamination, patients tend to a worse outcome [2]. We report the case of a subtle clinical presentation of PAEF in a 77-year-old man without history of abdominal aortic aneurysm or GI bleeding. The aim of this paper is to emphasize the insidiousness of the observed clinical presentation of PAEF and to stress the necessity for immediate treatment.

## CASE PRESENTATION

**Figure 1.**
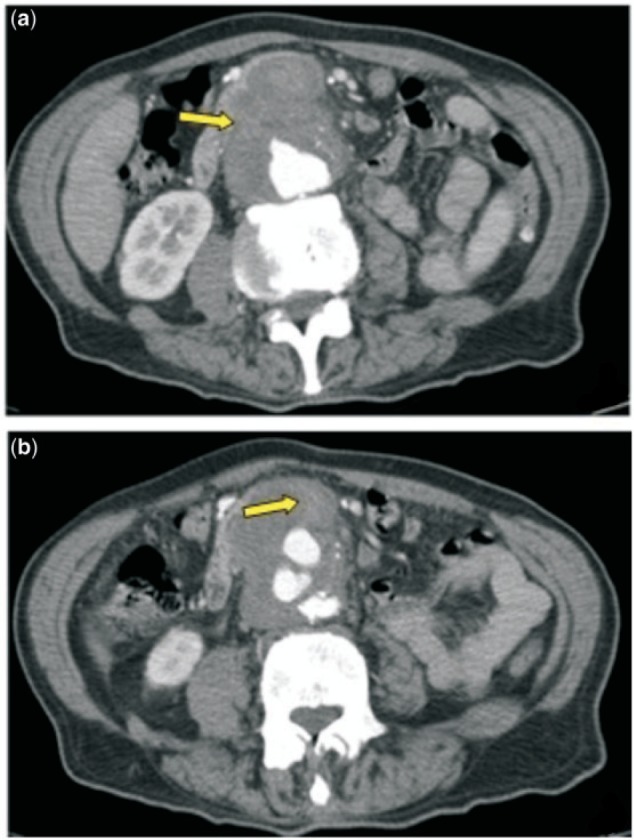
Enhanced CT scan; arrows showing: (a) absence of a clear separation between duodenal and aneurysmatic aortic wall; (b) fading contrast medium filling small bowel loop.

A 77-year-old Caucasian man consulted his general practitioner concerning a two-week history of worsening weakness and was referred to our emergency department upon detection of an abdominal bruit. At arrival, the patient was pale and diaphoretic; he was apyretic, eupneic, conscious and oriented (blood pressure 110/75; heart rate 85; respiratory rate 12; O_2_sat 98%). Physical examination confirmed a faint mesogastric bruit associated with an abdominal pulsatile mass. Melenic stool was found in the rectal vault on rectal examination. The patient had a medical history of myocardial infarction, hypertension and previous cigarette smoking, with no history of surgery. His treatment was acetylsalicylic acid and angiotensin-converting enzyme (ACE) inhibitor (enalapril). Blood tests showed leukocytosis (white blood cell 15 × 10^3^/uL), mild anaemia (haemoglobin 10.1 g/dL; hematocrit 32.2%) and alteration of the renal function (urea 93 mg/dL; creatinine 1.54 mg/dL). An electrocardiogram (ECG) confirmed previous anterior necrosis. An urgent esophago-gastroduodenoscopy (EGD) was arranged, but after a sudden drop in the patient’s blood pressure (60/45; heart rate 100), an enhanced abdominal CT was obtained first. The examination revealed a large (63 × 55 mm) thick-walled infrarenal aortic aneurysm, associated with distension of the small bowel loops by fluid content (Figure 1).

**Figure 2.**
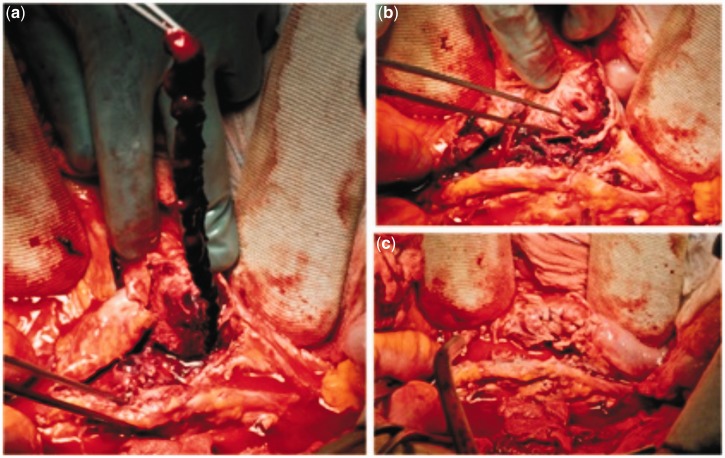
Intra-operative findings: (a) dissected aneurysmatic wall and long thrombus extracted from the aorto-duodenal fistula; (b) extraserous aspect of the duodenal fistula facing the aneurysmatic aorta; (c) duodenal suture.

The patient was immediately transferred to the operating theatre and a significant amount of fresh blood (400 mL) was aspirated through the nasogastric tube during anaesthetic preparation. The laparotomy disclosed a ruptured aneurysm of inflammatory appearance, tenaciously adherent to the fourth portion of the duodenum and surrounded by a small retroperitoneal haematoma, mainly confined to the paraduodenal area. Following aortic and iliac cross-clamping, dissection of the aneurysmatic aortic wall from the duodenum was performed and a 1 cm aorto-duodenal fistula was found upon removal of a fresh clot (Figure 2a and -b). The peritoneal cavity was irrigated with rifampicin, the duodenal wall was sutured with Vycril® (Ethicon, New Jersey) (Figure 2c) and the aorta was repaired by prosthetic interposition (bifurcated Intergard® silver knitted 16 × 8 mm), sutured with Prolene® 2-0. The aneurysmatic wall was closed above the prosthesis after collection of samples and a pedicle of omentum was interposed between the aortic wall and the duodenum.

On post-operative day (POD) one, the patient was re-operated for persistent anaemia and abundant blood loss into the abdominal drainage. Diffuse haemostasis was performed on the retroperitoneal para-aortic tissues with cauterization and sealants, in the absence of a relevant active haemorrhagic finding. The remaining post-operative course was uneventful. Histology of aortic wall samples confirmed an aspecific, chronic inflammatory pattern. Blood cultures were negative for bacterial or fungal infection; therefore a wide-spectrum antibiotic therapy with imipenem was administered for 30 days. On POD 15, the patient was transferred to a rehabilitation centre and discharged two weeks later. At 18 months' follow-up, the patient is fine and no evidence of infection was detected on imaging and laboratory tests.

## DISCUSSION

There is no consensus in dating the first finding of a PAEF by Ashley Cooper in the early nineteenth century; however the authorship of the term is attributed to him [3]. The first case report was published by Salmon in 1843 and, in spite of the passage of one hundred and seventy years, there are to date only about 400 similar cases in the literature [4].

PAEF is a rare occurrence, being the cause of less than 0.2% of all GI bleeding in a single series [5], and complicating only 0.7–2% of aortic aneurysms [6, 7]. Despite this, emergency physicians should raise their index of suspicion in patients with AAA. Indeed, PAEF occurs mostly in these patients (>80%), being responsible of almost 20% of GI bleedings in this specific population [8]. Degenerative aneurysms are preponderant over infective (especially Salmonella or Klebsiella ssp.) and traumatic aneurysms [9, 10]. Other reported causes of PAEF are peptic ulcer perforation, GI neoplasia infiltrating the aortic wall, tuberculous mesenteric lymphadenitis, primary aortitis, pancreatic pseudocyst penetration, ingested foreign body, diverticulitis, appendicitis and radiation injury [11–16].

Since the majority of aneurysms develop in the infra-renal portion of the aorta, the duodenum is the most frequent site of fistulization (>70%), mainly at the level of the third and fourth portions [2]. Other described sites of fistulization are the oesophagus, stomach and small and large bowels [17–19]. Cases of two co-existing PAEFs in the same patient have been reported [20].

Notably, the classic symptom triad of PAEF (GI bleeding, abdominal pain, and a pulsating abdominal mass) is displayed in only 10% of affected patients and clinical presentation is often subtile [8]; GI bleeding is almost invariably present, but the first few episodes are usually mild and self-limiting. In the beginning these so-called ‘herald bleeds’ may be the only manifestations of fistulization. Although one-third of patients experience massive recurrent bleeding within six hours [8], a higher proportion reports a long period (up to months) of sparse and self-limiting episodes. In these cases, PAEF is easily misinterpreted as a chronic GI bleeding, therefore being easily underestimated by patients or confused with peptic ulcer disease by physicians. Herald bleeds may even be so slight as to pass unnoticed until sudden occurrence of massive haemorrhage precipitates the patient into haemorrhagic shock [21].

Diagnostic work-up of suspected AEF, either primary or secondary, depends on patients’ haemodinamic condition. Haemorrhagically unstable patients mandate emerging laparotomy, therefore diagnosis is often obtained intra-operatively. The risk of recurrent bleeding immediately before surgical repair may be reduced maintaining blood pressure between 60 and 100 mmHg [22]. Stable patients with upper GI bleeding usually undergo EGD; however, quite often the third and fourth portion of the duodenum cannot be observed with traditional instrumentation (e.g. adults endoscope). Hence some series report low sensitivity of EGD in diagnosing AEF, with a 25% detection rate [23]; therefore, when CT scan was not available PAEF was detected incidentally by EGD. Specific findings include extrinsic compression of the distal duodenum, active bleeding or, in case of secondary AEF, direct visualization of aortic graft throughout the duodenal wall. The advent of enhanced CT has significantly improved prompt diagnosis of AEF, both primary and secondary. Even though enhanced CT detection proves a sub-optimal detection rate for PAEF (61%) [8], it can reveal all the associated common findings: AAA, absence of a clear separation between duodenal and aortic wall, signs of retroperitoneal inflammation, and peri-aortic air bubbles. The presence of contrast in the small bowel is pathognomonic of AEF.

Prompt treatment is essential. The mortality rate from surgery is 30–40% [8]. The surgical approach must consider the amount of retroperitoneal contamination, which is related to the bacterial flora and different pH levels, depending on the involved GI segment [24]. Inflammatory aneurysm (bacterial or mycotic)—as well as septic aortitis—although rare, must be considered as a source of contamination.

There is no consensus on the choice of the surgical approach, owing to the lack of large or long-term studies. Extra-anatomical bypass and repair of the enteric tract is the treatment of choice in cases of gross contamination; however this technique is associated with survival rates of 40–60% due to continuous haemorrhage from the aortic suture line [8], stump blow-out and rhabdomyolysis caused by prolonged cross-clamping. *In situ* reconstruction is often reported as an effective surgical option in case of mild bacterial contamination [25]. The reported survival rate for in-line reconstruction is 61–77% [26]. Most frequently, antibiotic-impregnated Dacron or PTFE graft are used. Cryopreserved arterial graft is a valid option when available, but higher costs and risk of aneurysmal degeneration or rupture are reported [27]. Interposition of omentum between the graft and the enteric suture line is appropriate [28]. Collection of tissue specimens for microbiological tests has facilitated targeted antibiotic therapies. *Escherichia coli*, *E. faecalis*, *salmonella*, *mycobacterium tuberculosis*, c*lostridium septicum*, *lactobacillus* and *Klebsiella* are most frequently isolated. Many authors suggest antibiotic therapy for at least one week if cultures are negative, whereas positive cultures demand extended administration for up to six weeks following surgery [1, 22].

Endovascular treatment has recently become a valid option. Unstable patients, particularly those with hostile abdomen, may benefit from prompt endovascular exclusion of the aortic fistula, in order to rapidly control the haemorrhage. Nevertheless, a staged approach with delayed surgical treatment seems widely to be advised; indeed, stent graft repair is associated with a high incidence of persistent, recurrent or new infection (44%). Furthermore, pre-operative evidence of infection is associated with poor outcome after endovascular repair [29]. Recurrent bleeding after endovascular treatment is also reported to occur frequently [30].

## CONCLUSIONS

Massive GI haemorrhage may be fatal. High suspicion for PAEF is vital in patients affected by AAA. Anaemia of unknown origin, worsening faintness, slight GI bleeding episodes or history of melenic stools must be always considered as potential signs of PAEF, until proven. In patients with unknown history of AAA, GI bleeding is rarely caused by PAEF, nevertheless clinical evaluation must be accurate in order to detect the presence of an abdominal bruit or pulsatile mass. In rare cases, EGD can lead to diagnosis. Enhanced CT has significantly improved PAEF detection; however, a definitive diagnosis is often obtained intra-operatively. Either *in situ* aortic replacement or extra-anatomical reconstruction, combined with debridement, are widely accepted treatments. Targeted antibiotic therapies should be continued after surgery. Endovascular repair can be effective for emergency treatment in haemorrhagic, unstable patients or severely compromised patients; however, exposure to graft infection mandates delayed definitive surgical procedure. Furthermore, acute contamination or delayed graft infection must be always taken into account.

*Conflict of interest statement:* none declared.
